# Comparative Metabolite Profiling of Wheat Cultivars (*Triticum aestivum*) Reveals Signatory Markers for Resistance and Susceptibility to Stripe Rust and Aluminium (Al^3+^) Toxicity

**DOI:** 10.3390/metabo12020098

**Published:** 2022-01-20

**Authors:** Manamele D. Mashabela, Lizelle A. Piater, Paul A. Steenkamp, Ian A. Dubery, Fidele Tugizimana, Msizi I. Mhlongo

**Affiliations:** 1 Research Centre for Plant Metabolomics, Department of Biochemistry, University of Johannesburg, P.O. Box 524, Auckland Park, Johannesburg 2006, South Africa; ngoatomd@gmail.com (M.D.M.); lpiater@uj.ac.za (L.A.P.); psteenkamp@uj.ac.za (P.A.S.); idubery@uj.ac.za (I.A.D.); Fidele.Tugizimana@omnia.co.za (F.T.); 2 International Research and Development Division, Omnia Group, Ltd., Johannesburg 2021, South Africa

**Keywords:** metabolomics, multivariate statistical analysis, primary and secondary metabolism, *Puccinia striiformis* f. sp. *tritici*, wheat

## Abstract

Plants continuously produce essential metabolites that regulate their growth and development. The enrichment of specific metabolites determines plant interactions with the immediate environment, and some metabolites become critical in defence responses against biotic and abiotic stresses. Here, an untargeted UHPLC-qTOF-MS approach was employed to profile metabolites of wheat cultivars resistant or susceptible to the pathogen *Puccinia striiformis* f. sp. *tritici* (*Pst*) and Aluminium (Al^3+^) toxicity. Multivariate statistical analysis (MVDA) tools, *viz*. principal component analysis (PCA) and hierarchical cluster analysis (HiCA) were used to qualify the correlation between the identified metabolites and the designated traits. A total of 100 metabolites were identified from primary and secondary metabolisms, including phenolic compounds, such as flavonoid glycosides and hydroxycinnamic acid (HCA) derivatives, fatty acids, amino acids, and organic acids. All metabolites were significantly variable among the five wheat cultivars. The *Pst* susceptible cultivars demonstrated elevated concentrations of HCAs compared to their resistant counterparts. In contrast, ‘Koonap’ displayed higher levels of flavonoid glycosides, which could point to its resistant phenotype to *Pst* and Al^3+^ toxicity. The data provides an insight into the metabolomic profiles and thus the genetic background of *Pst-* and Al^3+^-resistant and susceptible wheat varieties. This study demonstrates the prospects of applied metabolomics for chemotaxonomic classification, phenotyping, and potential use in plant breeding and crop improvement.

## 1. Introduction

Wheat (*Triticum aestivum* L.) is a stable dietary supplement for millions of people, providing nutrition in the form of vitamins, minerals, amino acids, and health-beneficial phytochemicals (e.g., phenolics) [1,2]. The primary sources of food produced from wheat include, but are not limited to, bread, pasta products, cereals, and baby food. In addition, some small quantities of wheat are also used for biofuel, animal feed, and alcohol production [3]. Wheat is the third most cultivated crop in the world after maize (*Zea mays*) and rice (*Oryza sativa*) in total production [4]. Though it originated from a natural hybridization of *Triticum urartu* and *Aegilops speltoides*, modern wheat is highly genetically diverse. Cultivation of wild ancestors and further hybridization ultimately gave rise to the economically important progenitor of bread wheat, known as *Triticum aestivum* L. [5].

Breeding programmes have further contributed to the genetic variability of wheat cultivars with the quest to enhance agronomic traits (i.e., improved growth, yields, disease resistance and abiotic stress tolerance). In addition, growth conditions and geographical locations can trigger modifications to the crop’s genome over time, resulting in a range of wheat cultivars with varying genetic and phenotypic traits [6,7]. Therefore, cultivar classification and screening for superior traits have been a primary focus in breeding programmes, especially with the rapid rate of climate change and the emergence of more virulent and destructive pathogens [8,9,10]. Furthermore, with rapid population growth (expected to be 9–10 billion people by 2050), the world has become increasingly urbanised. The food supply and demand ratio has significantly declined, placing pressure on food security globally [11]. Intensified and more efficient agricultural practices have coordinated efforts to close the gap and meet these ever-growing demands. However, food insecurity remains a significant concern for the world. More significantly, in Sub-Saharan African (SSA) nations, more than 240 million people live in extreme poverty and are subject to malnutrition and/or starvation [12,13]. The situation is further exacerbated by the prevalence of biotic and abiotic environmental stressors, such as stripe rust (*Pst*) and Aluminium (Al^3+^) toxicity that have ravaged primary crops in SSA.

*Pst* and Al^3+^ toxicity are especially concerning stressors on dryland wheat varieties due to high soil acidity, poor fertility, and nutrient stratification, as observed over the years [4,14]. Wheat stripe rust (*Pst*) is an economically important disease caused by the fungus *Puccinia striiformis* f. sp. *tritici* (*Pst*). The fungus poses a significant threat to wheat cultivations, resulting in 10 to 70% yield losses globally [15,16]. Al^3+^ toxicity also poses limitations on plant stress tolerance, resistance and development. Elevated concentrations of the toxic Al^3+^ produce reactive oxygen species (ROS). Subsequently, ROSs interfere with plant physiological processes, cell division, root elongation, nutrient and water uptake, plant growth, and overall crop production [17].

According to the Ref. [6], in recent years, agricultural and breeding experts from organisations such as the Agricultural Research Council for Small Grains (ARC-SG), Sensako and Pannar have worked on breeding programs to improve wheat cultivar varieties with regard to stress tolerance and disease resistance. The ARC-SG released improved wheat cultivars into the private sector for multiplication, leading to improved quality, productivity and increased yield quantities in the dryland growing region of South Africa [6,7]. Conventionally, plant breeders have relied on phenomics and plant phenotyping for the assisted selection of desired and superior traits for crop improvement, backed by genomic and proteomic data [18]. While gene expression profiling highlights differentially expressed genes, proteomic studies reveal the decrease or increase of proteins related to the expression of particular genes with little to no biochemical data [19]. However, such practices lack broader representation of genotype–environment (GxE) interactions. Metabolites are the most relevant to the phenotype and link the genomic and phenomics representations of plant responses to the environment [20]. The attraction to metabolomics studies is the ability to provide a direct link to transcriptomic and proteomic reprogramming and to display reliable biochemical markers through metabolites that are the immediate representations of the observable phenotype(s) [21].

The investigation of an organism’s metabolome, comprising non-protein small molecules, is a recent development in the “omics revolution”. The metabolomics approach qualitatively and quantitatively provides a rich, real-time source of information about an organism’s functional state [5,19]. By introducing metabolomics, breeders can decipher the underlying metabolic pathways, along with important intercellular regulatory biomarkers [22,23]. This knowledge could allow breeders to pinpoint essential metabolite-associated phenotypes for crop improvement based on interactions with the immediate environment [24]. In addition, metabolomic techniques have been used to analyse large-scale metabolic compositions and regulatory networks in plants, yielding novel biochemical knowledge that may be utilised in crop breeding and improvement to enhance desired traits.

The main objective of metabolomics in agriculture is to unravel and understand the metabolic responses of crops to environmental stressors for rapid and accurate phenotyping and breeding programmes [25]. Furthermore, metabolomics and chemometric tools can allow comprehensive grouping (based on similarity and differences) and classification of cultivars (based on biomarkers responsible for the observed phenotypes) [21,26]. This study compares the metabolic profiles of selected *Pst*-resistant and susceptible wheat cultivars. We also aimed to identify the key metabolites and biomarkers responsible for the differences in observed resistance or susceptibility in the cultivars under investigation. It was hypothesized that *Pst*-resistant cultivars would reveal accumulation of metabolites associated with an enhanced stress response, thus conferring resistance to the environmental stressors compared to the susceptible cultivars.

## 2. Results

### 2.1. Analysis of Chromatographic and Mass Spectrometric Data for Differential Detection of Metabolites in Wheat Cultivars

**Figure 1:**
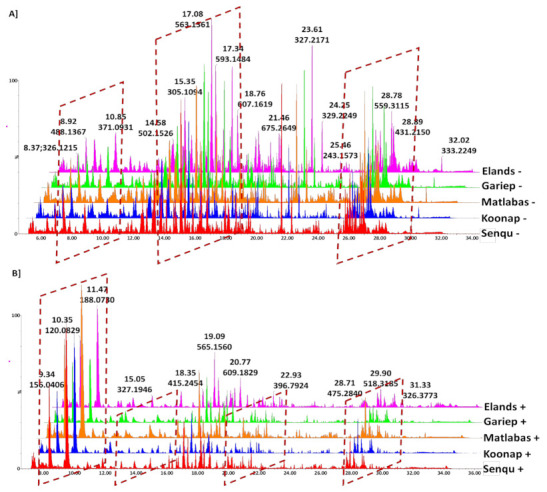
UHPLC-MS BPI chromatograms of methanolic extracts of wheat leaves. The chromatograms show comparative differences between the represented cultivars in the negative (**A**) and positive (**B**) ionisation modes. Visual inspection of the chromatograms clearly shows the quantitative and qualitative difference in the peak populations (presence/absence).

Crude methanolic extracts of leaf tissue from five dryland wheat varieties planted in winter–spring rainfall regions of South Africa (Table S1) were separated on ultra-high performance liquid chromatography coupled to a quadrupole time-of-flight high-definition mass spectrometry detector (UHPLC-qTOF-MS). Metabolite profiling was performed in both positive and negative electron spray ionisation (ESI) modes to allow inclusive, non-biased coverage and global analysis of the metabolome. The inspection of the extracted LC chromatograms displaying distinct base peak intensities (BPI) from negative Figure 1A and positive data Figure 1B showed good chromatographic separation considering the complexity of the multi-dimensional metabolome covering a broad range of polar and non-polar metabolites. Furthermore, the BPI MS chromatograms revealed differentially populated peaks (each with unique *m*/*z* values, intensities and retention times (Rts), representing the qualitative (presence/absence) and quantitative (intensity/concentration) detection of metabolites, thus providing a visual description of the similarities and differences between the selected wheat varieties.

BPI chromatograms showed better ionisation of metabolites in ESI^−^ mode, and thus, overall sensitivity (both qualitatively and quantitatively) of detected compounds as compared to the ESI^+^ mode. For plant phenolics and flavonoids, negative ionisation provides improved ionisation efficiency and the potential for lower detection limits [27]. However, both ionisation modes are significant for broad metabolite profiling and fingerprinting. A study by the Ref. [27] reported that ESI^−^ ionisation is preferred 46% of the time, generally by oxygen-based compounds, such as flavonoids and carboxylic acids. These compounds make up the majority of metabolites detected in this study. In contrast, ionisation of both oxygen-containing metabolites and nitrogen bases (e.g., amino acids) are more favourable in ESI^+^ mode, 38% of the time [21,27].

### 2.2. Chemometric and Statistical Data Analysis for Metabolite Profiling of Wheat Cultivars

**Figure 2:**
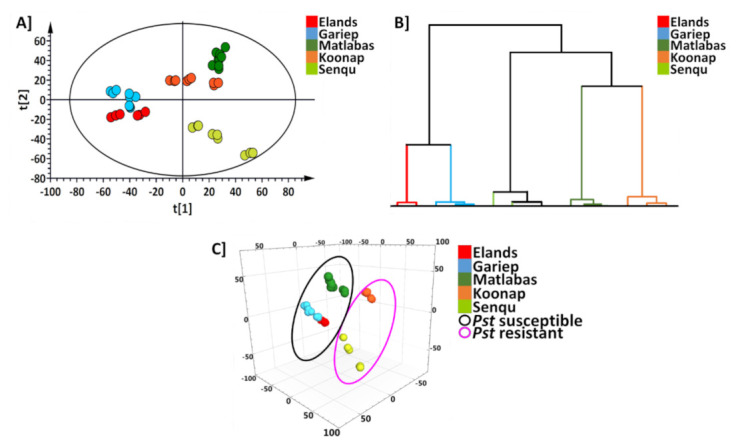
Unsupervised exploratory data analysis of ESI negative wheat cultivar classification data. (**A**) Principal component analysis (PCA) was based on the whole metabolomics dataset retrieved from the analysed wheat samples. The PCA shows discriminant sample clustering coloured by cultivars. The PCA was an 18-component model with R^2^X(*cum*) of 0.541 and Q^2^(*cum*) of 0.403. (**B**) An HiCA plot showing the hierarchical relations between the samples corresponding to (**A**). (**C**) A 3D PCA scores plot of the wheat samples; the 3D shows the discriminant clustering of the cultivars based on susceptibility (circled in blue) and resistance (circled in orange) to *Pst*. ‘Koonap’ (orange) represents the only cultivar resistant to both *Pst* and Al^3+^ toxicity, where it is chemically related to ‘Matlabas’, as seen by the closer clustering in both the PCA and HiCA. Furthermore, ‘Koonap’ is separated from all the other cultivars, as seen in (**C**), based on its metabolite profile, which could be a representation of its resistance to both types of stresses.

Due to the apparent differences observed on BPI MS chromatograms, statistical data analysis software and chemometric analysis were used to distinctly discriminate between the wheat varieties and establish a metabolite-based grouping/classification of the different cultivars. Data mining was performed through unsupervised principal component analysis (PCA), followed by multivariate data analysis (MVDA) to obtain PCA score plots with corresponding hierarchical cluster analysis (HiCA) for chemotaxonomic analysis, as graphically represented in Figure 2. Chemometric data analysis applies pattern recognition and machine learning algorithms to isolate significant features and trends otherwise not visible through visual inspections of the BPI MS chromatograms from complex data matrices for the biochemical systems under investigation, thus representing data in an interpretable manner [28]. The first and second principal components (PCs), (PC1 and PC2) explain 54.1% and 40.3% of the variation, respectively. The normalisation quality of the data was evaluated using quality control (QC) groupings to further investigate data reliability and evaluate the chromatographic and mass detection systems [29].

**Figure 3:**
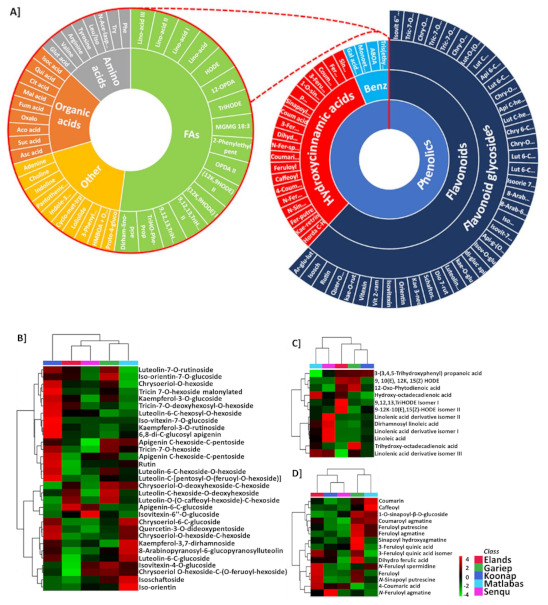
Distribution of annotated metabolites among five wheat cultivars. (**A**) The sunburst plot shows the classification of the annotated, putatively identified metabolites, and the main classifications of annotated metabolites were phenolics (with flavonoids and HCAs), fatty acids, organic acids and amino acids. (**B**–**D**) The distribution of selected annotated metabolites among five wheat cultivars according to class. Samples are projected in columns with the metabolites in rows. The data were log-transformed, and *Pareto* scaled in the Ref. [32]. Colour-coding indicates abundance (red = high abundance, green = low abundance). Some metabolites are found in high abundance in some cultivars, and very low abundance in other cultivars.

PCA separated the cultivars into five distinct groups (Figure 2A), showing differential clustering of the varieties based on their differences in metabolite profiles and distribution. It is important to note that differential clustering of the cultivars is not solely dependent on the qualitative composition of metabolites, but rather in combination with the differential quantitative distribution of each metabolite between the cultivars. The generated PCA score plot illustrates the similarities or differences within (intra-cultivar variance by PC2) and between (inter-cultivar variance by PC1) the sample clusters [30,31]. Figure 2A shows the grouping of *Pst*-susceptible cultivars together (‘Gariep’ and ‘Elands’), while the resistant cultivars of ‘Koonap’ and ‘Senqu’) were distributed further away from the susceptible cultivars. The ‘Matlabas’ cultivar is also susceptible to *Pst*; however, located towards the middle, separated from the ‘Gariep’ and ‘Elands’. The HiCA plot in Figure 2B complements this observation. The HiCA further reveals how the groups of cultivars chemically relate to or are separated from one another. The distinct separation of the five groups directly mimics the distribution of the groups as observed on the PCA, with ‘Senqu’ and ‘Koonap’ showing the most significant separation from ‘Elands’ and ‘Gariep’. The positioning of the ‘Matlabas’ cultivar closer to ‘Koonap’ suggests the composition of similar metabolic features associated with resistance as with the resistant cultivar, and this phenomenon is further visualised in the descriptive heatmap discussed below in Figure 3B.

The third principal component (PC3) was further evaluated to explore the patterns in the data with the 3D PCA model illustrated in Figure 2C. PC3 shows the closer grouping between the *Pst*-resistant cultivars (‘Koonap’ and ‘Senqu’—in purple demarcation), distinctively separating them from the *Pst*-susceptible cultivars (‘Elands’, ‘Matlabas’ and ‘Gariep’—in black demarcation). The exploration of data with applied PCA allowed for the reduction of the multi-dimensional data to a 2D model that was easier to interpret, thus revealing the underlying distribution patterns of the data. At the same time, the third PC added to the exposure of features seemingly obscured in the 2D model.

### 2.3. Evaluation of Differential Metabolic Profiles of Wheat Cultivars

Metabolite annotation and identification were concomitantly carried out using the BPI, and single-ion extracted chromatograms from samples of all five cultivars. Putative annotation of metabolites led to the identification of 100 metabolites spanning different metabolite classes originating from both primary and secondary metabolic pathways. A profile of these metabolites is graphically illustrated in the sunburst plot in Figure 3A. Table S2 lists the individual annotated metabolites, either commonly expressed within the five cultivars or present as unique biomarkers. Metabolite annotation was performed to level 2 of the Metabolomics Standard Initiative (MSI) based on product ion spectral information formed by collision-induced dissociation (CID) of selected parent ions [33]. Classes of positively identified metabolites included most phenolic compounds, such as flavonoids, hydroxycinnamic acid derivatives, and polyphenols. The list also consists of fatty acids, organic acids, and several amino acids (Figure 3A).

A hierarchical clustering interactive heatmap analysis (Figure 3B and Figure S2) was performed in the Ref. [33] to investigate the variations of the identified metabolites in the wheat cultivars using the average integrated peak areas of the respective metabolites. The heatmaps revealed unique diversities between the wheat varieties in terms of metabolite abundance or enrichment. For instance, the class of fatty acids was highly abundant in the resistant ‘Senqu’ cultivar, while many flavonoid glycosides were enriched in the resistant ‘Koonap’ variety. In contrast, enrichment of HCAs was observed in all susceptible cultivars (‘Matlabas’, ‘Elands’, and ‘Gariep’).

**Figure 4:**
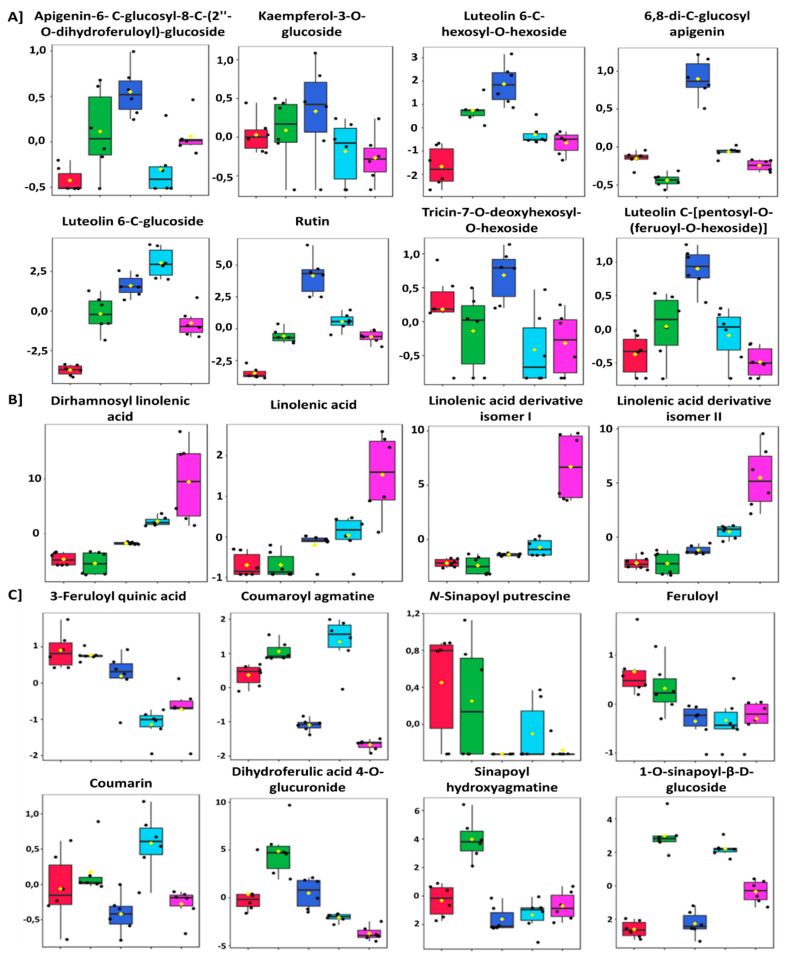
Box-and-whisker plots showing quantitative differences of metabolites per cultivar expressed as normalised concentration. The diagrams highlight the differential quantitative variations in the abundance of metabolites from the selected wheat cultivars per class. Yellow dots on the box and whiskers diagram represent the mean value and black dots indicate each replicate (single data points). (**A**) flavonoid glycosides, (**B**) fatty acids, and (**C**) hydroxycinnamic acid derivatives (from left to right of each plot: Eland = red, ‘Gariep’ = green, ‘Koonap’ = dark blue, ‘Matlabas’ = sky blue, and ‘Senqu’ = pink).

Figure 4 shows box-and-whisker plots representing an in-depth, semi-quantitative visualisation of the distribution of selected metabolites in the five cultivars. The inter-cultivar variations in metabolite profiles are illustrated by the increased or relative decrease of the mentioned metabolites. Figure 4A reveals relatively higher concentrations of flavonoid glycosides, such as rutin, kaempferol-3-O-glucoside, and luteolin-6-C-hexosyl-hexoside in the ‘Koonap’ cultivar, followed closely by both the ‘Matlabas’ and ‘Gariep’ cultivars. Surprisingly, ‘Senqu’ displayed a lower concentration of these metabolites, though expected to be closely related to ‘Koonap’ due to similar degrees of resistance to *Pst*. ‘Senqu’, however, has an accumulation of linolenic acid derivatives (Figure 4B). All three susceptible cultivars showed an interesting trend in the collective accumulation of HCAs, including feruloyl putrescine, feruloyl agmatine, coumaroyl agmatine and sinapoyl hydroxyagmatine, compared to their resistant counterparts (Figure 4C). The cultivar-based quantitative and qualitative metabolite distribution was further evidenced in the group-wise metabolite selection, biplot-based discrimination analysis, and the variable importance in projection (VIP) showing metabolic markers contributing to metabolic phenotypes in Figure S1A,B.

The general metabolite distributions observed in the hierarchical clustering interactive heatmap analysis and the box-and-whisker plots reveal the differential expression of metabolites per cultivar, thus giving insight into the variations in their metabolite profiles. Output from an orthogonal projection to latent structures-discriminant analysis (OPLS-DA), a supervised model for binary classification, displays clear discrimination between *Pst* and Al^3+^ toxicity-resistant (‘Koonap’) and susceptible (‘Gariep’) cultivars based on their respective metabolite profiles (Figure S3A). A receiver-operator characteristic (ROC) plot was used to evaluate the classification ability, sensitivity, and specificity of the OPLS-DA (Figure S3B), the ROC plot showed 100% sensitivity and specificity of the mode indicating the model’s high discriminatory power. A selection of features responsible for the observed cultivar classification was carried out on an OPLS-DA loading S-plot (Figure S3C), selecting only features with high correlation and covariation, [p(corr) ≥ 0.5, ≤−0.5 and (p1) ≥ 0.1, ≤−0.1] shown in blue and red demarcation, respectively. The relevance of the selected metabolites was further evaluated with a VIP score plot (Figure S3D) showing metabolites with VIP scores higher than the cut-off threshold of 1. Following the OPLS-DA analysis in ‘Koonap’ vs. ‘Gariep’, kaempferol-3-O-glucoside, dirhamnosyl linolenic acid, 6,8-di-C-glucosyl apigenin, and rutin (majority of flavonoids) were positively correlated to ‘Koonap’ and negatively correlated to ‘Gariep’. In contrast, examples of HCAs and fatty acids including trihydroxyoctadecenoic acid, 9,12,13, triHODE, dihydroferulic acid 4-O-glucuronide, and 1-O-sinapoyl-β-D-glucose had a positive correlation to ‘Gariep’ and negative correlation to ‘Koonap’. The above-mentioned metabolites contribute to cultivar-specific discrimination and differential metabolite profiles, which can serve as markers for *Pst* and Al^3+^ toxicity resistance.

**Figure 5:**
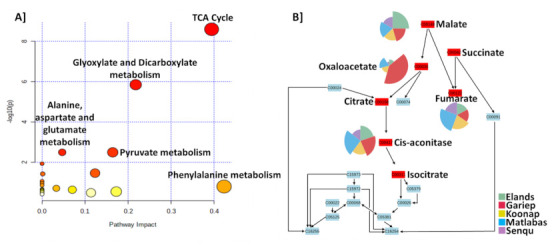
MetaboAnalyst-computed pathway analysis. (**A**) Pathway view of statistically significant pathways flagged from the metabolome view based on matched metabolites. The figure shows pathways matched from the annotated metabolites of all five wheat cultivars. The pathways are arranged based on the *p*-value (y-axis), which indicates the pathway enrichment analysis, and pathway impact values (x-axis) representing pathway topology analysis. The node colour of each pathway is determined by the *p*-value (red = lowest *p*-value and highest statistical significance), and the node radius (size) is based on the pathway impact factor, with the biggest indicating the highest impact. The phenylalanine metabolism, TCA cycle, and glyoxylate and dicarboxylate metabolism pathways were the most impactful among the different cultivars and showed the highest statistical significance (*p*-value < 0.05). (**B**) The diagram illustrates the integration and contribution of matched metabolites in the flagged TCA cycle pathway. The metabolites are matched using KEGG identifiers (e.g., citrate: C00158). The matched metabolites that function as TCA intermediates in the TCA cycle overlap with the glyoxylate and dicarboxylate metabolism pathways, which lead to the production of oxoglutarate. The pie charts indicate the abundance of each of the metabolites in the different wheat cultivars.

Chemometrically extracted metabolites in Table S2 were used to perform metabolic pathway analysis using MetaboAnalyst Pathway Analysis (MetPA) to reveal impactful metabolic pathways associated with the generated data across all five cultivars (Figure 5A). The phenylalanine (Phe), TCA cycle, glyoxylate and dicarboxylate metabolism, and pyruvate metabolism pathways showed relatively high impact based on matched metabolites from the data. The most statistically significant pathways were the TCA cycle, glyoxylate and dicarboxylate metabolism, pyruvate metabolism and the alanine, and aspartate and glutamate metabolism pathways listed above from the most significant and the most impactful pathways.

The TCA cycle functions as one of the most important metabolic pathways in plants, playing a role as the primary source of energy for cellular metabolism and the main link to almost all metabolic pathways. The metabolism of Phe channels carbon from photosynthesis to the phenylpropanoid pathway, which is involved in crucial functions, such as plant growth and development and the mitigation of biotic and abiotic stress. The TCA cycle intermediates malate, succinate, *cis*-aconitase, fumarate, citrate, iso-citrate and oxaloacetate were the most matched metabolites, showing the overlap between the most statistically significant pathways, the TCA cycle, and the glyoxylate and dicarboxylate metabolism pathways (Figure 5B). The pie charts illustrate the relative intensities of the metabolites in the five wheat cultivars.

## 3. Discussion

Biotic and abiotic stresses are the most investigated environmental factors associated with retarded plant growth, development, and, most importantly, commercial crop production and yields. Over the years, strategies have been developed and implemented to mitigate the deleterious effects of environmental stress on crops. Ultimately, agrochemical applications have been the method of choice for stress control; additionally, plant breeding has served as an alternative to producing biotic stress-resistant and abiotic stress-tolerant crops. The work presented herein was based on the hypothesis that genetic individuality of wheat cultivars/varieties confers uniqueness to metabolite profiles, enabling discrimination of resistant varieties from genetically distinct susceptible varieties by investigating individual metabolite variations in the different cultivars. In addition, such uniqueness in metabolite profiles could further determine the degree of resistance or susceptibility of wheat varieties to *Pst* and Al^3+^ toxicity.

Wheat is grown throughout heterogeneous areas across the globe, and varieties are specifically bred for resistance to different pathogens and insects and the adaptation to various agronomic conditions. Using well-established metabolomics techniques and chemometric statistical analysis tools, the metabolomes of five wheat cultivars were profiled. The identified metabolites showed variations, both qualitatively and quantitatively, among the different cultivars.

Secondary metabolites are some of the most ubiquitous metabolites in plants, the biogenesis of which occurs through the similarly ubiquitous phenylpropanoid pathway. According to the Refs. [34,35,36], cinnamic acids are the most common phenolic acids in wheat, including major derivatives of ferulic-, sinapic and coumaric acids. Amino acids identified included Phe, which in addition to functioning as building blocks of proteins, Phe plays a central role as a precursor molecule to several plant metabolites involved in growth development, reproduction, and defence [37]. Phe is crucial in channelling photosynthetic carbon to the biosynthesis of phenylpropanoid constituents. HCAs are synthesised early in the phenylpropanoid pathway through the deamination of Phe to *trans*-cinnamic acid that can be converted to *p*-coumaric acid, a known precursor compound to produce HBAs [37]. Phe was found in low abundance across all five cultivars, possibly indicating a negative correlation due to high conversion rates to its HCA derivatives.

The functions of phenolic compounds are attributed to their antioxidant properties. These capabilities span many physiological and adaptive processes in plants, such as morphological adaptations, reproduction, development and defense against biotic and abiotic environmental factors [38]. The antioxidant properties of phenolic compounds have been well-documented [21,38]. Flavonoid glycosides and HCAs are shielding compounds that protect plants from oxidative damage caused by reactive oxygen species (ROSs) by slowing down oxidative degradation and scavenging free radicals. These bioactive compounds have also been reported to display antimicrobial activity [39,40]. The mentioned traits have been attributed to the reducing properties of flavonoids and HCAs. Additionally, suppression of metal-catalysed free radicals has been reported [41], owing to their capabilities as metal-chelating compounds, particularly iron, copper, and Al^3+^, as measured in vitro [41,42]. Higher concentrations of flavonoid glycoside in ‘Koonap’ could point to the phenotypic resistance of the cultivar to *Pst* and Al^3+^. Interestingly, the ‘Matlabas’ cultivar, though susceptible to both *Pst* and Al^3+^, also displayed enhanced concentrations of flavonoid glycosides. ‘Matlabas’ is a winter wheat variety planted during the June season. According to the Ref. [43], winter wheat has a high tolerance to low temperatures. This observation can possibly be attributed to the enhanced levels of flavonoids glycosides present in the ‘Matlabas’ cultivar.

Further observation of the HCA heatmap shows an accumulation of HCAs in all the susceptible cultivars. It is important to note that although phenotypically, the cultivars are classified as either resistant or susceptible, particular metabolic features can be shared only with quantitative variations. Although suitable for spring cultivation, these cultivars have been subjected to the winter cultivation season. Hence, the HCA prevalence in these *Pst* and Al^3+^ susceptible cultivars may not necessarily be against the stresses mentioned above, but instead heightened for adaptive tolerance to the low winter temperatures. Additionally, the complexation behaviour of HCAs, such as caffeic, ferulic and *p*-coumarinc acid towards Al^3+^ cations have been reported [42]. The stable complex formation between Al^3+^ and HCAs is pH-dependent (pH 3.5 for caffeic acid, and pH 4.5 for both ferulic and *p*-coumaric acid) and forms due to the chelating capabilities of the carboxyl groups on the acids [42]. These reports are consistent with indications of high Al^3+^ toxicity in soils with high acidity [14], and thus highlight the potential role of HCAs to reverse or reduce effects of metal toxicity in plants. Furthermore, a study by the Ref. [44] reported that Al^3+^ stress triggers the accumulation of ferulic and *p*-coumaric acid in monocots such as rice. Results from the study also showed the roles of HCA compounds in Al^3+^ resistance through cell wall modifications (hemicellulose and lignin cross-linkage), thus disrupting the active binding of Al^3+^. The studies mentioned above could point to the possible adaptation of susceptible wheat cultivars to Al^3+^ toxicity.

There was also a clear separation between the ‘Koonap’ and ‘Senqu’ cultivars on the PCA and the HiCA plots. Both cultivars are resistant to *Pst*; however, the apparent differences in metabolite profiles may result from the differences in the resistance to Al^3+^ toxicity, which can be indicated by the lower levels of both flavonoid glycosides and HCAs in the Al^3+^-susceptible ‘Senqu’, as compared to the resistant ‘Koonap’. Besides salinity, Al^3+^ toxicity is one of the most widespread problems associated with ion toxicity in plants [45]. Phenolic compounds can reverse the effects of Al^3+^ toxicity on plants through metal complexing or chelating, as previously mentioned. These compounds, including flavonoid derivatives (glycosidic and sulphonate conjugates), form stable complexes with Al^3+^ for either internal root or shoot detoxification as metal antidotes. The phenolic or flavonoid-mediated resistance to Al^3+^ toxicity has been previously reported in maize [46,47].

According to the Ref. [48], phenolics present in wheat can be constitutive in nature. The efficiency of the phenolic metabolism was studied in wheat phenotypes resistant and susceptible to the Karnal bunt pathogen, *Neovossia indica*. Results showed onset elevation and activity of phenols in resistant phenotypes, followed by a significant decline by 10 days post-infection (dpi). In contrast, there was a slow accumulation of phenolics, such as caffeic acid, tyrosine, and hydroquinone in susceptible phenotypes, indicating delayed deployment and accumulation of these defence metabolites. The results suggest that both resistant and susceptible phenotypes can deploy similar defence mechanisms; however, the delayed deployment of the mechanism by susceptible phenotypes serves little purpose to prevent pathogen establishment. In this case, susceptible cultivars face early demise.

In contrast, early deployment of defence metabolites by resistant phenotypes ensures immediate resistance against the pathogen. Moreover, defence metabolites in resistant phenotypes do not play a role in ultimate resistance in the absence of disease [48,49]. As such, decreased biosynthesis or an accelerated catabolism of defence metabolites can serve alternative metabolic pathways; this phenomenon could explain the apparent down-regulation of flavonoid glycosides and HCAs in the ‘Senqu’ cultivar though phenotypically resistant to *Pst*. In addition, some of the primary roles of phenolic compounds function as structural components of plant cell walls. In maturing wheat grains, 75–80% of phenolic compounds occur in insoluble forms esterified to cell wall polymers, while 20–25% are esterified to sugars [35]. For instance, ferulic and *p*-coumaric acids identified in this study have been reported to esterify, with arabinose units of arabinoxylans functioning as components of cereal cell walls in wheat [50,51]. Additionally, phenolic acids such as HCAs and HBA derivatives play a vital role in the formation of secondary cell walls as precursors of monolignols in lignin biosynthesis [34].

A range of unsaturated fatty acids (UFAs) were identified from the wheat cultivars under investigation. According to the Ref. [52], the significant components of fatty acids in wheat include unsaturated oleic (18:1), linoleic (18:2), and linolenic (18:3) acids. UFAs are essential primary metabolites in cell maintenance as the functional constituents of the cell membrane and serve as precursors in the biosynthesis of phytohormones such as jasmonates, which are essential for plant responses to biotic and abiotic stress as signalling molecules [27,29]. Additionally, C18 UFAs are essential as signalling and regulatory molecules in the plant through functioning as hydrophobic hormones that bind to and regulate receptor proteins controlling major regulatory networks impacting cellular metabolism and signal transduction [53,54]. UFAs have been implicated in eliciting rapid stress response elements (RSREs), which respond to various kinds of biotic and abiotic stress [54]. For instance, increased levels of both 18:2 and 18:3 unsaturated FAs were found to confer resistance to *Colletotrichum gloeosporioides* in avocado and *Pseudomonas syringae* in tomatoes [55]. FAs were more enriched in the ‘Senqu’ cultivar, followed by ‘Gariep’, indicating the possible role of FAs against *Pst*. Moreover, the Ref. [56] reported the processes of lipid metabolism as markers for induced resistance in wheat. These findings could explain the positive correlation of UFAs in the *Pst*-resistant ‘Senqu’ cultivar.

Organic (carboxylic) acids (OAs) and amino acids were present in different quantities in the varying wheat cultivars among the identified metabolites. Carboxylic acids and amino acids are generally constitutive in nature [27,57]. Carboxylic acids are involved in primary metabolism as intermediates of the TCA cycle responsible for energy production in the cell, osmoregulation, as well as plant growth and development. Additionally, TCA intermediates are good signalling molecules; their ability to form complexes and the ease of metabolism in different cell compartments allow them to reflect the metabolic and redox state of the cell [58]. According to the Ref. [59], organic acids such as malate, citrate, succinate, acetate, oxalate, and tartrate chelate with active cations of Al^3+^ form inactive complexes of OA-Al^3+^. The non-toxic OA-Al^3+^ complexes are generally formed in the rhizosphere and thus reduce Al^3+^ uptake or accumulation, further limiting metal toxicity [59,60]. Furthermore, Al^3+^-induced efflux of OAs has been demonstrated to be a major resistance mechanism in several plant species, including wheat [61,62]. These activities can span several metabolic pathways. The identified carboxylic acids are involved in the pyruvate metabolism and are synthesised as a result of the Ala, Asp and Glu metabolism, further showing the versatility of the compounds in plant metabolism. Functions of carboxylic acids can be extended to precursors of amino acid biosynthesis [20,27].

Overall, the outcomes of the study revealed that significant metabolite variations in organisms could be both qualitative and quantitative, as many metabolites were common in all five cultivars, with variations in enrichment. Metabolic differences in the wheat varieties resulted in apparent cultivar-associated differences based on genetic differences between the cultivars. Genetically related wheat cultivars can vary in metabolite compositions and distribution attributed to the phenotype and functional agronomical properties. Additionally, some discriminant metabolic markers were identified from the selected wheat cultivars. For example, quinic acid and valine were identified as metabolic markers for ‘Senqu’, along with linolenic acid and its derivatives. At the same time, iso-orientin and 9,12,13 triHODE were identified as markers for ‘Koonap’ and trihydroxy-octadecenoic acid and (10E,15Z)-9,12,13-trihydroxy-10,15-octadecadienoic acid for ‘Elands’ and ‘Matlabas’, respectively. The differential regulation and accumulation of these metabolites in the wheat varieties could ultimately determine their growth parameters and metabolic responses to environmental factors. Moreover, metabolic markers can be useful for the selection of functionally favourable phenotypic and agronomical traits for breeding practices.

Plant breeding provides the opportunity to reduce the cost of disease control and management, as well as losses in crop productivity and yields, thus providing substantial economic benefits for primary crop producers. Even so, crops showing susceptibility to the stressors mentioned above are still utilised. The investigation of an organism’s metabolome is a recent development in the “omics revolution” and provides a rich, real-time source of information about an organism’s functional cellular state.

This study constitutes an attestation of the ability of metabolite profiling to drive hypothesis generation through the identification of plant metabolites and potential pathways of metabolite biosynthesis that distinguish between wheat cultivars. These findings support the use of global high-throughput metabolite profiling as a discovery tool capable of identifying a specific pattern or ‘profiles’ responsible for traits of interest in plants under investigation. This study is only one part of a more significant effort to develop sustainable wheat production by elucidating the metabolite profiles of wheat cultivars relative to their traits of resistance and vulnerability to environmental stressors.

## 4. Materials and Methods

### 4.1. Wheat Cultivation

Five wheat cultivars (namely, ‘Elands’, ‘Matlabas’, ‘Koonap’, ‘Senqu’ and ‘Gariep’) with varying degrees of resistance and susceptibility to *Pst* were obtained from the Agricultural Research Council for Small Grains (ARC-SG) in Bethlehem, Free State Province, South Africa (Table S1). The seeds were grown in potted and autoclaved germination mix (Culterra, Muldersdrift, South Africa) under controlled greenhouse conditions: minimum temperature 15 °C and maximum temperature of 25 °C. Plants were watered twice a week with distilled water and fertilizer mixture consisting of 650 mg/L CaNO_3_, 400 mg/L KNO_3_, 300 mg/L MgSO_4_, 90 mg/L mono-ammonium phosphates, 90 mg/L mono-potassium phosphates, 150 mg/L Soluptase, 20 mg/L Microplex, and 40 µL/L Kep-P-Max obtained from Shiman SA (Olifantsfontein, South Africa). Cultivar variants were grown in three biological replicates (three separate pots per cultivar).

### 4.2. Plant Harvesting and Metabolite Extraction

Leaves were harvested from the plants at the three-leaf stage, snap-frozen, and then stored at −80 °C until extraction. Metabolite extraction was carried out as follows: 1 g of leaves were pulverised in liquid nitrogen, followed by resuspension in 10 mL 80% ice-cold methanol. The solution was sonicated twice with a probe sonicator at 55% power for 30 s each time, at room temperature, followed by centrifugation at 5000 rpm for 15 min at 4 °C. The supernatant was collected and evaporated to approximately 1 mL at 55 °C with a rotary evaporator (Heidolph, Schwabach, DE). The extracts were dried to completeness in a vacuum microcentrifuge at 46 °C, then reconstituted in 300 µL of 50% LC-grade methanol (Romil, Cambridge, UK) and filtered through 0.22 μm nylon syringe filters into 2 mL HPLC vials fitted with a 300 µL insert. Samples were stored at 4 ℃ until analysis. Quality control (QC) samples were prepared by pipetting equal volumes of the samples in a designated LC-MS vial for analysis.

### 4.3. Metabolomics-Based Data Acquisition, Analysis, and Interpretation

#### 4.3.1. Untargeted Metabolomics Study with UHPLC-MS Analysis

Metabolomics data acquisition was performed on 2 µL of 50% methanol extracts on a Waters Acquity UHPLC system fitted with an ACQUITY UHPLC HSS T3 column (1.8 µm × 2.2 mm × 150 mm) at a column temperature range of 60 °C (Waters, Manchester, UK), hyphenated to a SYNAPT G1 q-TOF-MS (Waters Corporation Milford, MA, USA). Each sample representing a biological replicate was analysed in triplicate technical replicates. A binary mobile phase was composed of water (eluent A) and acetonitrile (eluent B) (Romil Pure Chemistry, Cambridge, UK), both with 0.1% formic acid and 2.5% isopropyl alcohol (Sigma-Aldrich, Munich, Germany), at a flow rate of 0.4 mL/min and a run time of 30 min. The gradient was as follows: eluent B ranged from 2% over the first 2.0 min, 2–90% over 2.0–25 min, 90–95% over 25–27 min, then returned from 95–2% over 28–30 min. Finally, the column was washed with a solution of methanol:acetonitrile: isopropyl alcohol (MeOH:ACN: IPA) for regeneration after each batch analysis.

Sample ionisation was carried out in an ESI source in both positive and negative modes on a Waters SYNAPT G1 q-TOF MS. The MS conditions were set as follows: 2.5 kV capillary voltage and 30 V sample cone voltage with a 1800 V MCP detector voltage, a source temperature of 120 °C, and a 450 °C desolvation temperature. The cone gas flow was set at 50 L/h, desolvation gas flow at 550 L/h, *m*/*z* range of 50–1200, a 0.1 s scan time in centroid mode with interscan delay: 0.02 s, and a mass accuracy window of 0.5 Da. The MS was set to perform both unfragmented and five fragmenting experiments (MS^E^) simultaneously by increasing in-source collision energy from 3 to 30 eV to assist with subsequent structural elucidation and compound identification [29]. MassLynx^TM^ 4.1 software (SCN 704, Waters Corporation Milford, MA, USA) was used to regulate the LC-MS run. Sample analysis was carried out in three technical replicates to account for analysis variability. Pooled (QC) samples were included in the analysis to assess the reliability and reproducibility of the analytical method [63,64].

#### 4.3.2. Data Processing and Multivariate Data Analysis (MVDA)

UHPLC-qTOF-MS raw data were pre-processed by MarkerLynx^TM^ software (version 4.1, Waters Corporation, Milford, MA, USA) for both positive and negative data. The MarkerLynx^TM^ multistep data pre-processing allows for peak picking, retention time correction and alignment, noise elimination, feature detection and sample normalisation, creating data matrices of retention time (Rt)-*m*/*z* variable pairs, with *m*/*z* peak intensity for each sample [64]. MS data were processed as per the following parameters: Rt range of 0.68–26.35 min, a mass range of 100–1200 Da, a mass tolerance of 0.05 Da, and noise elimination level of 10, followed by data normalisation on MassLynx XM^TM^ software (Waters, Manchester, UK). For MVDA, processed data matrices were exported for analysis on SIMCA 14.0 software (Umetrics, Umea, Sweden). PCA, an unsupervised statistical analysis method, and orthogonal-partial least square discriminant analysis (OPLS-DA), a more supervised machine learning data analytical method, were performed, both in *Pareto* scaling for variable normalisation. The resulting PCA reduced the dimensionality of the data to present summarised indices from the data matrix for better visualisation and interpretation. In addition, OPLS-DA score plots were used for binary classification to reveal underlying metabolite features contributing to the observed discrimination between data groups.

Metabolomic diagnostic tools available in SIMCA were used to evaluate the generated models. These include the cumulative (*cum*) variation model in the matrix *X*, R^2^X (*cum*) and R^2^Y (*cum*), which explain the goodness-of-fit parameters, the response variable’s fraction of variance, and the predictable fraction of the matrix X by the assessed components. CV-ANOVA (analysis of variance testing of cross-validation predictive residuals) was used to validate the OPLS-DA data. Furthermore, a good OPLS-DA model was represented by a *p*-value of < 0.05. The OPLS-DA S-plot highlighted discriminant biomarkers. Further statistical evaluation of the model was carried out using receiver operator characteristic (ROC) and variable importance in projection plot (VIP) score plots. Annotation of significant ions contributing to the variation in the composition of the different cultivar varieties as depicted in the OPLS-DA scores was performed as described [30,65]. Compound annotation was done on MS-based accurate mass and fragmentation patterns. Existing spectral information was used to compare different collision energies of single ion extracted chromatograms (XICs) from each significantly induced ion. These were then compared to published data. Corresponding precursor ions were used to calculate empirical formulas, which were then used to search databases such as Dictionary of Natural Products [66], PubChem [67], and ChemSpider [68] for putative MSI (Metabolomics Standards Initiative) level 2 compound identification [31,69].

## 5. Conclusions

Breeding practices provide plant resistance at a genomic and molecular level, further conferring protection through epigenetic and transcriptomic modifications. Hence, many transgenic crops, such as wheat varieties displaying traits of resistance and tolerance to environmental stressors, including *Pst* and Al^3+^ toxicity, have been introduced into the fields through breeding programmes. The application of LC–MS-based metabolomics in this study allowed for the elucidation of the differential metabolite features of metabolomes of wheat cultivars and has provided a gateway to classification methods through metabolite profiling. The untargeted LC–MS approach led to the identification of a range of metabolites belonging to four major classes of phenolics (flavonoids and HCAs), fatty acids, organic acids, and amino acids. Furthermore, results from this study suggest that both qualitative and quantitative factors contribute to the discrimination of sample groups. The identification of significant metabolic markers can further reveal a plant’s robust arsenal in combatting that mentioned previously, and other biotic and abiotic stress or strategies employed in growth and development. Knowledge of the characteristics mentioned above can enhance research into plant breeding practices for crop improvement.

## Data Availability

Publicly available datasets were analyzed in this study. This data can be found here: [http://www.ebi.ac.uk/metabolights/MTBLS1234].

## Supplementary Materials

The following are available online at https://www.mdpi.com/article/10.3390/metabo12020098/s1, Figure S1: Distribution of significant metabolites. (**A**) A bi-plot metabolite distribution per cultivar and (**B**) VIP scores. Figure S2: Distribution of annotated metabolites (organic acids and amino acids) among five wheat cultivars, Figure S3: Supervised OPLS-DA statistical analysis and significant feature extraction of ESI negative data of methanolic extracts from leaf tissues, Table S1: Dryland wheat varieties sourced from different summer rainfall regions. Table S2: Summary of the annotated, putatively identified metabolites (MSI-L2) from 5 wheat cultivars.
