# Supplementary material for: Comparative Metabolite Profiling of Wheat Cultivars (Triticum aestivum) Reveals Signatory Markers for Resistance and Susceptibility to Stripe Rust and Aluminium (Al3+) Toxicity
Source: Metabolites. 2022 Jan 20;12(2):98. doi: 10.3390/metabo12020098 (PMC8877665; doi:10.3390/metabo12020098)
Supplement: Supplementary file 1 [file metabolites-12-00098-s001.zip › metabolites-1499432-supplementary.pdf]

metabolites

MDPI

Supplementary material

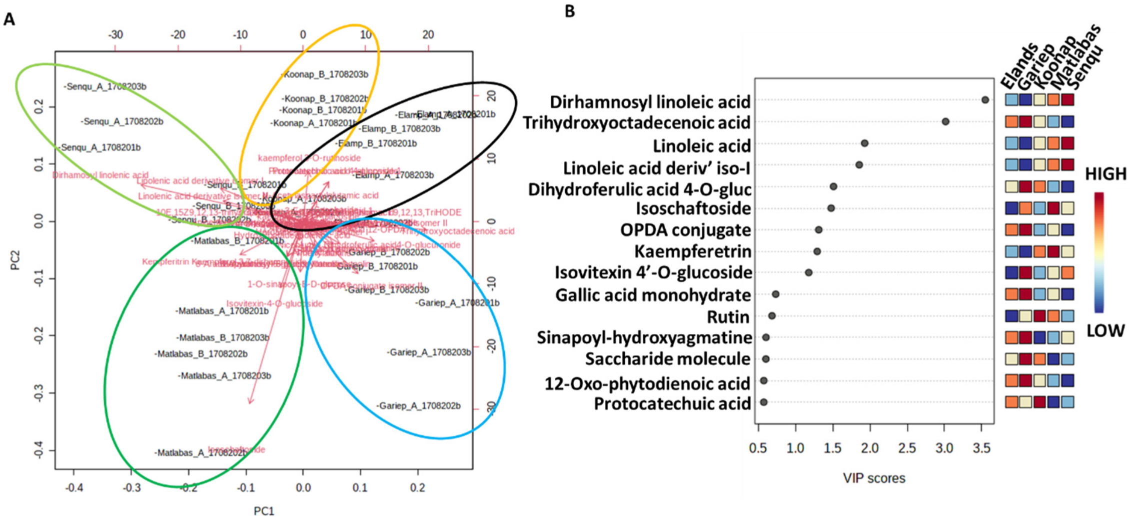

Figure S1. Distribution of significant metabolites. (**A**) A bi-plot metabolite distribution per cultivar and (**B**) VIP scores. The VIP scores plot revealed discriminating metabolite features from the classes of fatty acids and phenolics to play essential roles in the differential clustering of the wheat varieties in **A**. The coloured boxes to the right of the VIP score indicate the relative concentration of each metabolite in the respective cultivars, and the represented metabolites possess a VIP score of 0.5 or higher. The most discriminating metabolites originated from the fatty acids and phenolic classes possessing a VIP of 1 and above, with dirhamnosyl linoleic acid presenting the highest VIP score of 3.5.

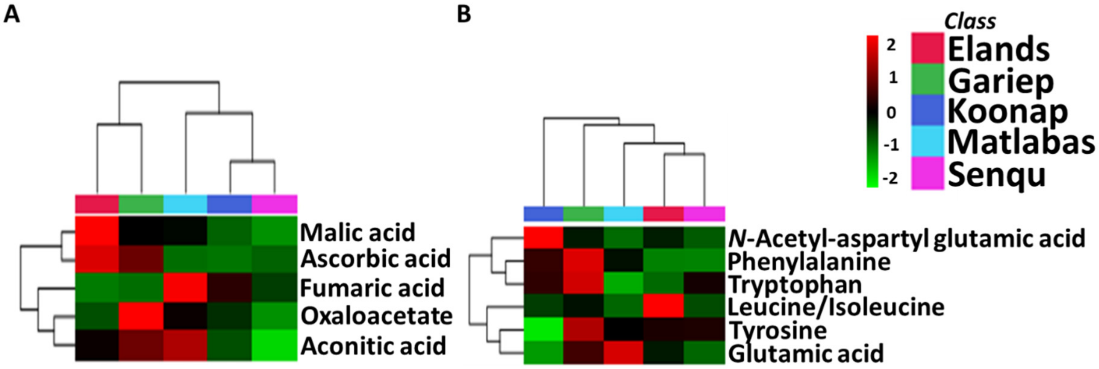

Figure S2. Distribution of annotated metabolites among five wheat cultivars: **A** and **B** shows the distribution l;0of selected annotated metabolites among five wheat cultivars according to class. Samples are projected in columns with the metabolites in rows. The data was *Pareto* scaled in [32]. Colour coding indicates abundance (red = high abundance, green = low abundance). Some metabolites are found in high abundance in some cultivars and very low abundance in other cultivars.

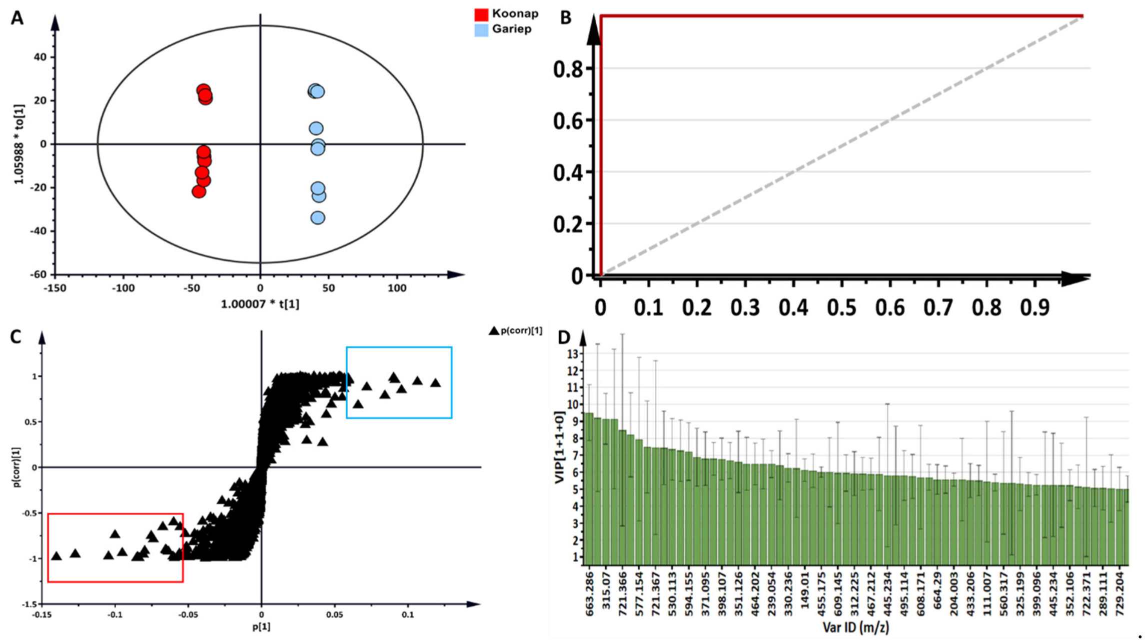

Figure S3. Supervised OPLS-DA statistical analysis and significant feature extraction of ESI negative data of methanolic extracts from leaf tissues (**A**): An OPLS-DA scores plot of respective *Pst* and Al^{3+} susceptible and resistant 'Gariep' *vs.* 'Koonap' cultivars. The model represented is 1+1+0 components with R^2X of 0.464, R^2Y of 0.999 and $Q^2(cum)$ of 0.985. (**B**): A receiver operator characteristic (ROC) plot was used to evaluate the sensitivity and specificity of the OPLS-DA, the ROC shows 100% sensitivity and specificity of the model. (**C**): An OPLS-DA S- plot corresponding to the model in **A** and shows discriminating variables between the selected cultivars (demarcated in red and blue rectangles) identified as significant discriminant variables. The significance of the variables selected in (**C**) was evaluated with the VIP score plot (**D**), with only variables displaying a VIP score of more than one considered significant.

Table S1. Dryland wheat varieties sourced from different summer rainfall regions. The wheat varieties used in this study display varying characteristics of susceptibility and resistance to stripe rust caused by *Puccinia striiformis* f. sp. *tritici* (*Pst*) and Aluminium toxicity.

Variety	Dryland wheat varieties for the summer rainfall region			
	**Planting date**	**Growth-type**	***Pst***	**Aluminium toxicity**
Elands	July	Intermediate	Moderately susceptible	Susceptible
Matlabas	June	Winter	Susceptible	Susceptible
Koonap	July/August	Intermediate	Resistant	Resistant
Senqu	July	Intermediate	Resistant	Susceptible
Gariep	July	Intermediate	Susceptible	Susceptible

Table S2. Summary of the annotated, putatively identified metabolites (MSI-L2) from 5 wheat cultivars. The qualitative and quantitative distribution of the metabolites is depicted in the heatmap (Figure 2B), and the classification of the metabolites is graphically represented in the sunburst plot (Figure 2A).

No.	Compound	Chemical Formula	Rt (min)	Theoretical mass	Experimental mass	Ionisation mode	Parent ion	Fragments (*m/z*)
**1**	L-Arginine	C6H15N4O2	0.75	175.1189	175.1025	Positive	[M+H]+	158, 130, 118, 116
**2**	Choline	C5H14NO	0.86	104,1708	104.1062	Positive	[M+H]+	60,
**3**	Adenine	C5H5N5	0.92	135.1267	136.0658	Positive	[M+H]+	118,
**4**	Citraconic acid	C5H6O4	0.98	130.0987	129.0194	Negative	[M-H]-	85
**5**	Malic acid	C4H6O5	1.04	134.0874	133.0127	Negative	[M-H]-	191, 115
**6**	Quinic acid	C7H12O6	1.24	192.1666	191.0182	Negative	[M-H]-	173, 111
**7**	Aconitic acid	C6H6O6	1.28	174.1080	173.0889	Negative	[M-H]-	129, 117, 85
**8**	Indoline	C8H9N	1.85	119.1638	120.0814	Positive	[M+H]+	103,
**9**	L-Phenylalanine	C9H11NO2	2.03	165.1891	164.0706	Negative	[M-H]-	147, 129, 103
**10**	Protocatechuic acid-4-glucoside	C13H16O9	2.18	316.2620	315.0755	Negative	[M-H]-	153, 108
**11**	Pantothenic acid	C9H17NO5	2.23	219.2350	220.1169	Positive	[M+H]+	185, 116, 90
**12**	Alanylleucine	C9H18N2O3	2.54	202.2540	203.1404	Positive	[M+H]+	157, 132, 86
**13**	Indole-3-carboxaldehyde	C9H7NO	2.98	145.1580	146.0616	Positive	[M+H]+	118, 91
**14**	3-(3,4,5-Trihydroxyphenyl) propanoic acid	C9H10O5	3.20	198.1740	197.0412	Negative	[M-H]-	153, 151, 138, 123, 109
**15**	L-Valine	C5H11NO2	3.35	117.1463	118.0868	Positive	[M+H]+	70, 55
**16**	L-Tryptophan	C11H12N2O2	3.37	205.0971	205.0968	Positive	[M+H]+	188, 146, 118
**17**	L-Leucine~~/Isoleucine~~	C6H13NO2	3.87	132.8054	132.0865	Positive	[M+H]+	86, 69
**18**	L-Glutamic acid	C5H9NO4	4.19	147.1293	148.0580	Positive	[M+H]+	130, 84, 72
**19**	Coumarin	C9H6O2	4.19	146.1450	147.0470	Positive	[M+H]+	103, 91
**20**	L-Tyrosine	C9H11NO3	4.24	181.1885	182.0807	Positive	[M+H]+	165, 136, 147, 123, 119, 91
**21**	Feruloyl agmatine	C15H22N4O3	4.71	306.3602	307.1698	Positive	[M+H]+	307, 273, 177, 145
**22**	Ferulic acid	C10H9O3	4.82	194.1840	177.0552	Positive	[M+H]+	145, 117, 89
**23**	Cyclomethyltryptophan	C12H12N2O2	5.08	216.2359	217.0977	Positive	[M+H]+	144,
**24**	Feruloyl putrescine	C14H20N2O3	5.19	264.3202	265.1552	Positive	[M+H]+	248, 177, 145
**25**	Tri (ethyl carbonate)	C16H18O11	5.37	386.1547	385.0782	Negative	[M-H]-	297, 89
**26**	*N*-Feruloyl sperm~~a~~idine	C17H28N3O3	5.77	321.2177	322.1654	Positive	[M+H]+	321, 177, 163, 146, 117, 89
**27**	3-Indole acrylic acid	C11H9NO2	5.80	187,1947	188.0712	Positive	[M+H]+	146, 144, 118, 102, 72
**28**	3-Feruloyl quinic acid	C17H20O9	5.91	368.1107	367.0970	Negative	[M-H]-	351, 219, 193, 178, 134, 102
**29**	3-Feruloyl quinic acid isomer	C17H20O9	5.92	368.3353	367.1045	Negative	[M-H]-	193, 134
**30**	Coumaroyl agmatine	C14H20N4O2	6.24	276.3342	277.1623	Positive	[M+H]+	260, 218, 147, 145, 131, 114

**31**	2-O-Glucosyl-7-methoxy-1,4(2H)-benzoxazin-3-one (HMBOA + O-Hex)	C15H19NO9	7.17	357.3127	356.0978	Negative	[M-H]-	300, 194, 166, 138
**32**	Saccharide compound	C16H20O10	7.29	372.3200	371.0976	Negative	[M-H]-	249, 231, 121, 113
**33**	Nicoblumin	C25H42O13	7.31	550.6000	549.2547	Negative	[M-H]-	387, 227
**34**	*N*-Feruloyl agmatine	C15H22N4O3	8.05	306.3602	307.1725	Positive	[M+H]+	290, 248, 247, 178, 177, 145, 117, 114, 95
**35**	*N*-Acetyl-aspartyl glutamic acid	C11H16N2O8	8.06	304.2550	303.0820	Negative	[M-H]-	303, 96
**36**	4-acetyl-2(3H)-Benzoxazolone (ABOA)	C9H7NO3	8.11	177.1568	178.0497	Positive	[M+H]+	150, 122, 95, 86
**37**	Sinapoyl hydroxyagmatine	C16H24N4O5	8.59	352.1470	351.1268	Negative	[M-H]-	249, 101
**38**	Luteolin-6-C-hexoside-O-hexoside	C27H30O16	8.88	610.5200	611.1612	Positive	[M+H]+	449, 451, 413, 329
**39**	Dihydroferulic acid 4-O-glucuronide	C16H20O10	9.25	372.3240	371.0978	Negative	[M-H]-	195, 175
**40**	Luteolin-C-hexoside-C-pentoside Isomer	C26H28O15	9.61	580.1428	579.1350	Negative	[M-H]-	489, 459, 399, 369, 339
**41**	1-O-Sinapoyl-β-D-glucose	C17H22O10	9.81	386.1213	385.1135	Negative	[M-H]-	223, 164
**42**	8-Arabinosyl-6-glucosylluteolin	C26H28O15	9.96	580.4915	579.1332	Negative	[M-H]-	561, 489, 459, 399
**43**	Kaempferol-3-O-galactoside-7-O-rhamnoside	C27H30O15	10.11	594.5181	593.1525	Negative	[M-H]-	447, 283
**44**	Luteolin-6-C-hexosyl-O-hexoside	C27H30O16	10.15	610.1585	611.1700	Positive	[M+H]+	593, 575, 545, 461, 431, 413, 395, 383, 353, 329, 299
**45**	Kaempferol-3-O-rutinoside	C27H30O15	10.45	594.5181	593.1498	Negative	[M-H]-	447, 300, 285, 284
**46**	Isovitexin-7-O-glucoside	C27H30O15	10.45	594.5181	593.1506	Negative	[M-H]-	473, 431, 341, 311,
**47**	Quercetin-3-O-pentosyl-pentoside	C25H26O15	10.46	566.5000	565.1477	Negative	[M-H]-	447, 309, 285
**48**	Hordatine-C-hexose isomer I	C44H33O16	10.52	772.2131	771.2019	Negative	[M-H]-	771, 609, 593, 503, 473
**49**	Rutin	C27H30O16	10.54	610.5175	609.1469	Negative	[M-H]-	593, 447, 309, 285
**50**	Apigenin C-hexoside-C-pentoside	C26H28014	10.60	564.4921	565.1557	Positive	[M+H]+	547, 529, 511
**51**	Schaftoside	C26H27014	10.61	564.4921	565.1670	Positive	[M+H]+	427, 409,379, 337, 325
**52**	Sinapoyl aldehyde	C11H12O4	10.84	208.2106	209.0797	Positive	[M+H]+	181, 177, 121
**53**	Isoschaftoside	C26H28O14	10.86	564.4921	563.1401		[M-H]-	473; 353; 325
**54**	Luteolin-C-hexoside-O-deoxyhexoside	C27H30O15	10.86	594.5181	595.1663	Positive	[M+H]+	449, 431, 383, 353, 329, 299
**55**	Luteolin-6-C-glucoside	C21H20O11	10.86	448.3800	447.0917	Positive	[M+H]+	431, 413, 353, 329, 299

**56**	Kaempferol-3-neohesperidoside	C27H30O15	10.87	594.5000	595.1775	Positive	[M+H]+	449, 299, 229,
**57**	Iso-orientin	C21H20O11	11.16	448.3769	447.0927	Negative	[M-H]-	429, 357, 327, 285
**58**	Apigenin-6-C-glucosyl-8-C-(2''-O-dihydroferuloyl)-glucoside	C34H28O21	11.37	772.5830	771.2049	Negative	[M-H]-	593, 503, 473, 383
**59**	Kaempferol-3-O-glucoside	C21H20O11	11.40	448.3769	447.0907	Negative	[M-H]-	285, 284, 255, 227
**60**	4-Coumaric acid	C9H8O3	11.44	164.1580	165.0531	Positive	[M+H]+	145, 123, 119, 103, 89, 69
**61**	Loliolide	C11H16O3	11.58	196.2429	197.1178	Positive	[M+H]+	179, 161, 133, 107
**62**	6,8-di-C-glucosyl apigenin	C27H30O15	11.62	594.5181	593.1506	Negative	[M-H]-	575, 473, 372
**63**	Chrysoeriol-O-hexoside-C-hexoside	C28H32016	11.78	624.5480	625.1769	Positive	[M+H]+	463, 445, 427, 409, 397, 367, 343, 313
**64**	Apigenin-6-C-glucoside	C21H20O10	11.84	432.1056	433.1135	Positive	[M+H]+	415, 397, 379, 349, 337, 313, 283
**65**	Kaempferol-3-O-rhamnoside-7-O-rhamnoside	C27H30O14	11.91	578.1635	577.1519	Negative	[M-H]-	431
**66**	Vitexin-2''-O-rhamnoside	C27H30O14	11.93	578.5187	579.1659	Positive	[M+H]+	433, 415, 397, 367, 313, 204
**67**	Isovitexin	C21H20O10	11.93	432.3775	433.1105	Positive	[M+H]+	415, 397, 367, 313, 204
**68**	Kaempferitrin	C27H30O14	12.13	578.5230	577.1557	Negative	[M-H]-	563, 453, 431, 413, 355, 341, 293, 283
**69**	Chrysoeriol-O-deoxyhexoside-C-hexoside	C28H32O15	12.28	608.5000	609.1819	Positive	[M+H]+	463, 445, 427, 409, 397, 367, 343, 313
**70**	Chrysoeriol-6-C-glucoside	C22H22O11	12.28	462.1162	463.1240	Positive	[M+H]+	445, 427, 409, 397, 391, 379, 367, 343, 313
**71**	Chrysoeriol-O-hexoside	C22H22O11	12.29	462.1162	463.1240	Positive	[M+H]+	301
**72**	Diosmetin-7-rutinoside	C28H32O15	12.31	608.5447	609.1820	Positive	[M+H]+	463,
**73**	3-Phenyl lactic acid	C19H10O3	12.37	166.1739	165.0552	Negative	[M-H]-	147, 119, 103, 73, 59
**74**	*p*-Coumaraldehyde	C9H8O2	12.42	148.1590	147.0430	Negative	[M-H]-	119,103, 59
**75**	Isoorientin-7-O-glucoside	C27H30O16	13.01	610.5175	611.1570	Positive	[M+H]+	449, 431, 383, 353, 329, 299
**76**	Luteolin-C-[pentosyl-O-(feruoyl-O-hexoside)]	C36H36O18	13.02	756.1900	757.2074	Positive	[M+H]+	449, 431, 413, 309, 177
**77**	Chrysoeriol-O-hexoside C-(O-feruoyl-hexoside)	C38H40O19	13.02	800.7140	801.230	Positive	[M+H]+	463, 445, 117
**78**	Tricin-7-O-deoxyhexosyl-O-hexoside	C29H34O16	13.22	638.6120	639.1925	Positive	[M+H]+	493, 331
**79**	Tricin-7-O-hexoside	C23H24O12	13.57	492.4102	493.1330	Positive	[M+H]+	331
**80**	Gallic acid monohydrate	C9H16O4	13.71	188.1300	187.0951	Negative	[M-H]-	169, 125

**81**	Caffeoyl ~~ic acid~~	C9H7O3	13.85	163.0395	163.1123	Positive	[M+H]+	145, 135, 117, 89
**82**	Isovitexin-6''-O-glucoside	C27H30O15	14.11	594.5100	595.1630	Positive	[M+H]+	595, 433, 415, 367, 337, 313, 283
**83**	Tricin-7-O-hexoside malonylated	C26H26O15	14.55	578.4770	577.1300	Positive	[M+H]+	493, 331
**84**	Luteolin-O-(O-caffeoyl-hexoside) C-hexoside	C36H36O19	15.58	772.6700	773.1929	Positive	[M+H]+	449, 431, 329
**85**	(10E,15Z) 9,12,13-trihydroxyoctadeca-10,15-dienoic isomer I	C18H32O5	17.27	328.4436	327.2171	Negative	[M-H]-	229, 211, 183, 171, 113
**86**	Trihydroxyoctadecenoic acid	C18H34O5	17.93	330.5000	329.2296	Negative	[M-H]-	229, 211
**87**	9-Hydroxy-12-oxo-10(E),15(Z)-octadecadienoic acid isomer I	C18H30O4	18.07	310.4340	309.2078	Negative	[M-H]-	291, 197
**88**	(10E,15Z)-9,12,13-Trihydroxy-10,15-octadecadienoic acid	C18H32O5	19.00	328.4490	327.2015	Negative	[M-H]-	307, 291, 227, 213, 209, 185, 155
**89**	Linolenic acid derivative isomer III	C30H38O3	20.05	446.6000	445.2310	Negative	[M-H]-	311, 293, 277
**90**	12-Oxo-phytodienoic acid (12-OPDA)	C18H28O3	20.12	292.4131	291.1910	Negative	[M-H]-	273, 247, 209, 165
**91**	OPDA conjugate isomer II	C18H30O4	20.14	310.4000	309.1913	Negative	[M-H]-	291, 273, 247, 209, 165
**92**	*N*-Sinapoyl putrescine	C15H22N2O4	20.76	294.3462	295.2245	Positive	[M+H]+	207, 175, 147, 119
**93**	9-Hydroxy-12-oxo-10(E),15(Z)-octadecadienoic acid isomer II	C18H32O3	20.96	296.4000	295.2256	Negative	[M-H]-	291, 247, 165
**94**	Linolenic acid	C18H30O2	21.12	278.4296	277.2149	Negative	[M-H]-	253, 235, 221, 197, 183, 179, 161, 113, 89
**95**	Linolenic acid derivative isomer I	C33H56O14	21.13	676.8000	675.3592	Negative	[M-H]-	415, 397, 277, 235, 89
**96**	Linolenic acid derivative isomer II	C33H56O14	21.42	676.8000	675.3744	Negative	[M-H]-	415, 397, 277, 235, 89
**97**	Arachidonic acid	C20H32O2	21.66	304.4669	305.2474	Positive	[M+H]+	121
**98**	Monogalactosylmonoacylglycerol (MGMG 18:3)	C27H46O9	22.28	560.3103	559.3075	Negative	[M-H]-	513, 277, 253, 235
**99**	Dirhamnosyl linolenic acid	C28H48O11	22.48	560.3124	559.3118	Negative	[M-H]-	277
**100**	Hydroxy octadecadienoic acid	C18H32O3	22.69	296.4510	295.2273	Negative	[M-H]-	277, 233, 195
